# NINJ1 regulates plasma membrane fragility under mechanical tension

**DOI:** 10.21203/rs.3.rs-5237916/v1

**Published:** 2024-10-16

**Authors:** Yunfeng Zhu, Fang Xiao, Yiling Wang, Yufang Wang, Jialin Li, Dongmei Zhong, Zhilei Huang, Miao Yu, Zhirong Wang, Joshua Barbara, Christopher Plunkett, Mengxue Zeng, Yiyan Song, Zhongxing Wang, Changjie Cai, Xiangdong Guan, Scott Hammack, Liang Zhang, Feng Shao, Zheng Shi, Fu-li Xiang, Jie Xu

**Affiliations:** 1 Department of Critical Care Medicine, First Affiliated Hospital of Sun Yat-sen University, Guangzhou, China; 2 Institute of Precision Medicine, First Affiliated Hospital of Sun Yat-sen University, Guangzhou, China; 3 Department of Chemistry and Chemical Biology, Rutgers, The State University of New Jersey, Piscataway, NJ, USA; 4 Institute of Intelligent Manufacturing Technology, Shenzhen Polytechnic University, Shenzhen, China; 5 Novartis Biomedical Research, San Diego, CA, USA; 6 National Institute of Biological Sciences, Beijing, China; 7 Department of Anesthesiology, First Affiliated Hospital of Sun Yat-sen University, Guangzhou, China; 8 NHC Key Laboratory of Assisted Circulation and Vascular Diseases, Sun Yat-sen University, Guangzhou 510080, China; 9 Corresponding author; 10 Lead contact; 11 These authors contributed equally

## Abstract

Plasma membrane integrity is vital not only for cell survival but also nearly all aspects of cell functioning^1^. Mechanical stress can cause plasma membrane damage^2^, but it is not known whether there are large molecules (proteins) that control plasma membrane integrity. Here we constructed a 384-well cellular stretch system that delivers precise, reproducible mechanical strain to adherent cells. Using the system, we screened 10,843 siRNAs targeting 2,726 multi-pass transmembrane proteins for stretch-induced membrane permeability changes. The screen identified NINJ1, a protein recently proposed to regulate pyroptosis and other lytic cell death^3,4^, as the top hit. We demonstrate that NINJ1 is a critical regulator for mechanical force-induced plasma membrane rupture (PMR), without the need of stimulating any cell death programs. Low NINJ1 expression renders the membrane more resistant to stretching, while high expression of NINJ1 lowers the threshold of PMR under mechanical strain. NINJ1 level on the plasma membrane is inversely correlated to tension required to rupture the membrane. In the pyroptosis context, NINJ1 on its own is not sufficient to fully rupture the membrane, and additional mechanical stress is required for full PMR. Our work establishes that NINJ1 functions as a *bona fide* determinant of membrane biomechanical properties. Our study also suggests that PMR across tissues of distinct mechanical environments is subjected to fine tuning by differences in NINJ1 expression and external mechanical forces.

Plasma membrane is the physical barrier separating the internal contents of the eukaryotic cells from the outside. It also serves as a structural support to house the membrane associated proteins, including sensors, receptors, channels, transporters and cytoskeletal components that carry out essential functions for the homeostasis of the cell^1^. Damage to the plasma membrane compromise its integrity and leads to the ionic imbalance, loss of chemical and electrical gradient across the membrane, leakage of cellular contents and in severe cases, cell death^5,6^. Mechanical stress is one of the major contributors of plasma membrane damage^2^. Physiological processes such as locomotion and resistance exercise cause microscopic tears on the membrane. This type of light damage activates an array of receptors, including the ion channels and G-protein coupled receptors, to initiate the downstream signaling pathways for repair and remodeling^7^. On the other hand, severe membrane damage, caused by pathological events such as trauma, exposure to penetrating sound waves, etc., may trigger inflammatory responses, leading to the recruitment of immune cells and the release of cytokines and chemokines^2,5,8^. On the cellular level, external forces, including compression, stretch and fluid shear stress induce strain on the cellular membrane, cause membrane permeability changes through mechanically activated channels and pore-forming protein complexes, disruption in lipid bilayer and cytoskeleton, damage to membrane-associated proteins and in extreme cases, membrane rupture^8–12^. Lytic cell death, which involves a full rupture of the plasma membrane, is the end point to most forms of cell death, including necroptosis, apoptotic necrosis, pyroptosis, etc^13^. The disintegration of plasma membrane and the resulting release of large-sized cellular contents, known as damage associated molecular patterns (DAMPs), to the surroundings is instrumental for the recruitment of immune cells, development of inflammation, initiation of repairs or clean up. Mechanical strains have been implicated in affecting the plasma membrane integrity in these processes^5^. However, how mechanical forces influence the plasma membrane rupture on the cellular level, and how it is regulated on the molecular level, is not fully clear. New insights in this area will facilitate the identification of therapeutic targets for disease condition associated with membrane damage, as well as the development of the strategies to mitigate cellular damage, promoting repair, modulate inflammation and immune response, etc.

## Construction of the high-throughput cellular stretch system

**Extended Data Fig. 1:**
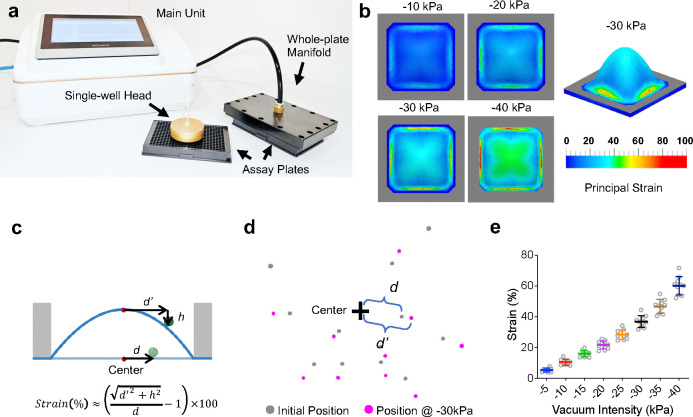
The mechanical characterization of the high-throughput cellular stretch system. **a**, The image of the HT cellular stretch system. The main unit features a touch-screen user interface. It connects to the either a vacuum head for single well use on inverted microscopes, or a whole-plate manifold for use with fluorescence plate imagers for high-throughput experiments. **b**, Finite element analysis of the PDMS membrane under different vacuum levels showed the strain pattern across the whole well. The strain pattern was largely uniform in the center, with slightly higher strain in the corners. The edges showed significantly higher strain, but it is outside of the measuring area of most plate imagers, therefore does not impose a problem in a real-world screening exercise. **c**, Illustration of the bead imagery assay utilized to empirically estimate the strain values with the formula shown. **d**, The bead images before stretch (gray) and at −30 kPa (magenta) were overlayed. *d* is the distance of the bead from the center of the well at the resting state, and *d*’ is the distance of the same bead from the center in the stretched state. **e**, Estimated strain values at different vacuum levels from the bead imagery analysis.

**Fig. 1:**
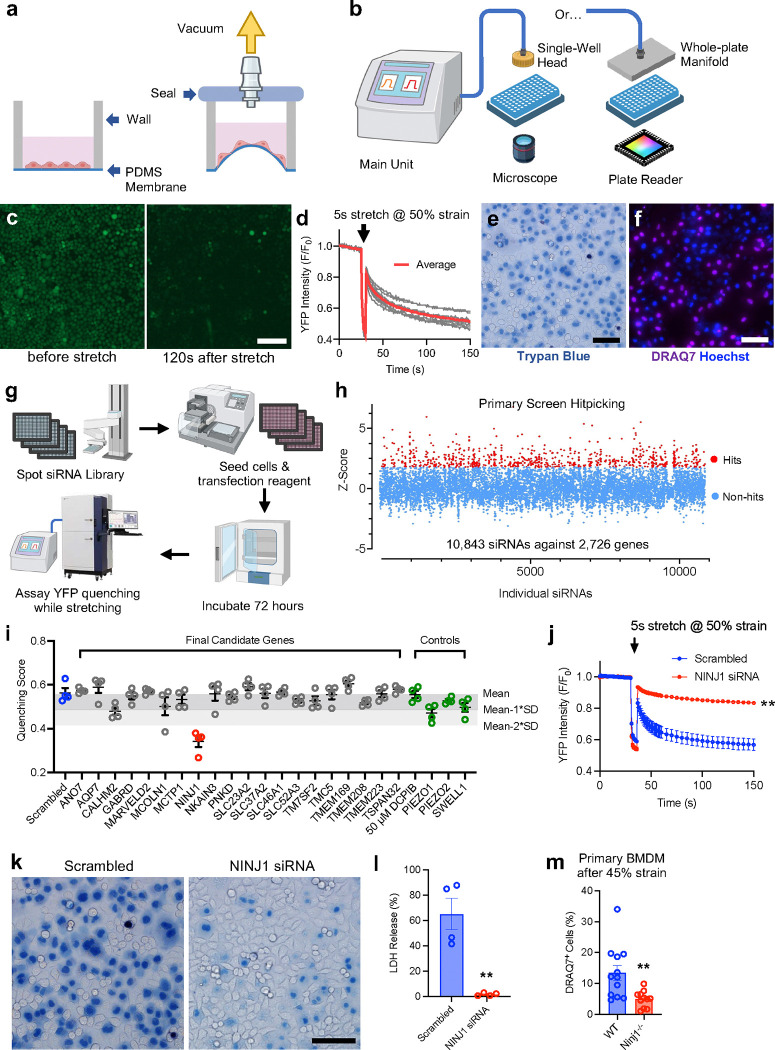
High-throughput genetic screen identifies NINJ1 as a regulator of plasma membrane rupture induced by mechanical stretch. **a**, The operating principle of the HT stretch system. Adherent cells are cultured on the optical quality PDMS membrane on the bottom of the multi-well assay plate. The well is sealed from the top. When vacuum is applied, the PDMS membrane bulges into the well and expands, applying mechanical strain to the cells adhered on top. **b**, The system was designed to be modular and easy to configure for either microscope use for one well at a time, or for a plate reader to record all wells simultaneously. **c**, HeLa cells stably expressing anion-sensitive YFP displayed fluorescence reduction after the application of mechanical stretch of 50% strain. Image shown were from immediately before and 120 s after the application of stretch. Scale bar, 150 μm. **d**, The YFP intensity decreased to half of the original level at 120 s after stretch. Each trace was from average fluorescence of one well from the 10 wells assayed. **e**, **f**, Post-stretch trypan blue and DRAQ7/Hoechst staining showed that ~50% of the cells had compromised plasma membrane. Scale bars, 100 μm. **g**, The workflow of the siRNA screen for genes regulating stretch-induced membrane damage. **h**, Scattered plot showed the overview of the primary screen covering 2,726 genes encoding multi-pass transmembrane proteins. Each dot was the data from one individual well corresponding to one of the 10,843 siRNAs used. Red dots were primary hits using >1.5 Z-score as the cutoff. Primary hit rate is 6.69%. **i**, The last round of reconfirmation with the 20 final candidate genes and a selection of 4 control genes showed that NINJ1 was the sole hit of this screen. **j**, Fluorescence quenching of HeLa-YFP cells induced by a 5 s stretch at 50% strain. Cells were transfected with a pool of 4 siRNAs against NINJ1 or scrambled control and stretched 72 h after. n=4 trials for each group. **k**, Trypan Blue staining of control and NINJ1 knockdown cells showed reduced plasma membrane rupture events induced by mechanical strain. Scale bar, 100 μm. **l**, LDH release from the control HeLa cells and the HeLa cells with NINJ1 knocked down. n=4 trials for each group. **m**, The percentage of DRAQ7^+^ primary BMDMs from *Ninj1*^−/−^ mice and WT littermates after the application of 5 s stretch at 45% strain. Each data point is from 100~200 cells in one of the stretched wells. For each genotype, primary BMDMs were isolated and pooled from 4 animals. Unless otherwise noted, all data are mean ± s.e.m.. ** p<0.01 vs respective control groups.

To identify genes important for regulating plasma membrane integrity under mechanical tension, we undertook the approach of high-throughput (HT) genetic screening. Previously we have demonstrated the success of this approach in identifying novel receptors sensitive to fluid shear stress using a custom HT flow stimulation system^14^. However, that particular system does not generate enough shear stress to break the plasma membrane. Therefore, we opted to design and construct an HT mechanical stimulation system for our needs with three specific standards: high-throughput capability, robust strain generation, and good compatibility with commercial screening hardware and robotics automation systems. We centered our design concept on the optical-quality PDMS stretchable substrates and the means to attach such substrates to commercially available bottomless polystyrene assay plates. We also devised the use of vacuum applied via a sealable lid to stretch the PDMS membrane, which in turn creates a state of negative pressure in the wells, thus stretching the cells adhered on top of the flexing membrane surface (Fig. 1a). The response of the cells could be monitored with from the bottom of the plate, either by a microscope or an image reader, in forms of fluorescence or luminescence changes. We designed our system to be modular with a main unit housing the vacuum pump, electronic valves, sensors and controllers, connecting either to a vacuum head for single well use, or a full-plate manifold for stretch all 384 wells simultaneously (Fig. 1b, Extended Data Fig. 1a). Finite element analysis (FEA) of the silicone membrane deformation under various vacuum levels showed that the stretch system generates strain levels higher than 50% with moderate vacuum of −40 kPa (Extended Data Fig. 1b). To empirically measure the strain generated under various vacuum levels, we performed fluorescent particle imaging with the system set at different vacuum levels ranging from −5 to −50 kPa (Extended Data Fig. 1c, d). We found that our system can induce 5%~60% strain with vacuum varying from −5 to −40 kPa (Extended Data Fig. 1e) and overall, these values are in agreement with the FEA analysis. For a movie of the system in operation, see Supplementary Video 1.

## Characterize the cellular response to mechanical strain

**Extended Data Fig. 2:**
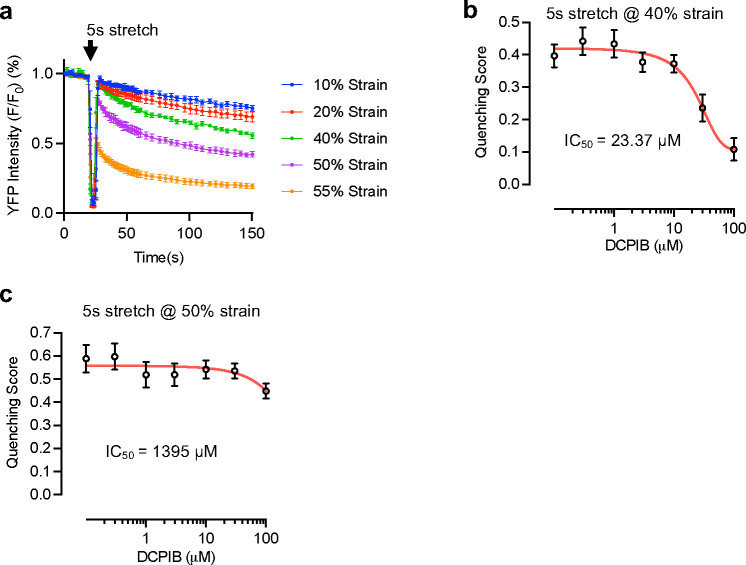
Characterization of quenching response of HeLa-YFP stable cells to mechanical strain. **a**, HeLa-YFP cells were subjected to stretches of 5s duration at a series of strain levels. Data are mean ± s.e.m.. Each trace is from 150~200 cells from n=3 to 4 wells per group. **b**, The inhibition curve of the DCPIB, a non-specific chloride channel inhibitor, on the quenching of YFP induced by 5s stretch at 40% strain. Each data point is presented as the mean ± s.e.m. from ~200 cells of each condition. **c**, The inhibition curve of the DCPIB on the quenching induced by 5s stretch at 50% strain. Each data point is presented as the mean ± s.e.m. from ~220 cells of each condition. Half-inhibition concentration at both strain levels were derived from the curves and noted.

We reasoned that when the integrity of plasma membrane is compromised under stretch, the permeability of the plasma membrane increases and the extracellular anions (e.g., chloride, etc.) will enter the cells, either through ion channels, pores or microscopic tears on the membrane. Therefore, we chose the highly sensitive YFP quenching as the primary assay for the screen. In brief, a mutated version of YFP with elevated sensitivity to anions, which reduces its fluorescence intensity upon binding chloride or iodide due to decreased the quantum yield and shifted the emission spectrum, was used to visualize anion level changes inside the cell^15^. We engineered a HeLa cell line stably expressing anion-sensitive YFP, and monitored the fluorescence while applying a 5-second pulse of stretch. We found that at moderate levels of stretch, i.e., 40% strain or lower, mechanical stretch led to a decrease in YFP fluorescence intensity. The reduction in fluorescence is dependent on the amplitude of the strain applied (Extended Data Fig. 2a, the sharp decrease of the fluorescence during the application of the stretch is an artifact resulting from cells going out of focus). The YFP quenching was inhibited by non-specific chloride channel inhibitor DCPIB in a dose-dependent manner, indicating that under these conditions, the anions enter the cells through ion channels (Extended Data Fig. 2b). At 50% strain, the quenching was robust and reaches half of original intensity at 120 seconds post-stretch (Fig. 1c, d, Supplementary Video 2). DCPIB was not able to inhibit the quenching at this strain level (Extended Data Fig. 2c), suggesting that the anions enter the cells via alternative paths, likely through microscopic tears or crevices on the plasma membrane formed under mechanical tension. Trypan blue staining post-stretching showed a significant percentage of cells with blue signal in the cytoplasm (Fig. 1e). Cells were also positively stained with DRAQ7, a chemical dye that cannot enter cytoplasm unless there are tears on plasma membrane (Fig. 1f). Taken together, these results suggest that HeLa-YFP cells experienced mechanically-induced plasma membrane rupture events at 50% strain. We therefore choose this strain level as the optimal stimulus in the following genetic screen for potential regulators of plasma membrane integrity.

## siRNA screening for genes regulating plasma membrane integrity under mechanical stretch

To reduce the amount of work required, we opted to construct a focused siRNA library instead of using a genome-wide one. We reasoned that the proteins that have the most impact on the plasma membrane permeability and integrity are likely the membrane-integrated proteins. Through bioinformatic means, we identified the 2,897 genes that have two or more predicted transmembrane segments, cross-referenced them with commercially available siRNAs, and generated a list of 10,843 siRNAs against 2,726 genes (see Supplementary Information for the full list). We then arrayed the library on to 384-well stretch plates, with one siRNA per well, then seeded the cells with transfection reagent, and assayed stretch-induced YFP quenching after 72 hours of incubation (Fig. 1g). For each well, the fraction of reduction in YFP fluorescence, termed Quenching Score, was derived and used as the primary readout, and then we calculated the deviation of the quenching score of each well from the mean of all data points, termed Z-score (see Methods for detailed data processing work flow). A cut-off Z-score > 1.5 was used for the primary screen, resulting in 755 hits, or a primary hit rate of 6.96% (Fig. 1h). We conducted two additional rounds of reconfirmation screen, gradually tightening the cut-off parameters and shortening the candidate list. In the final round, we obtained a list of 20 final candidate genes, and tested them with siRNAs purchased from a separate source, and identified NINJ1 as the sole hit of this screen (Fig. 1i). Note that knocking down the mechanically-activated ion channels PIEZO1 and PIEZO2, and the volume regulated anion channel SWELL1 has no impact on stretch-induced YFP quenching under high, rupture-inducing strain conditions, suggesting that those well-known channels in cellular mechanosensation and volume regulation are likely not the major regulatory contributors of strain-induced membrane damage (Fig. 1i)^15,16^.

## NINJ1 regulates plasma membrane rupture under mechanical tension

**Extended Data Fig. 3:**
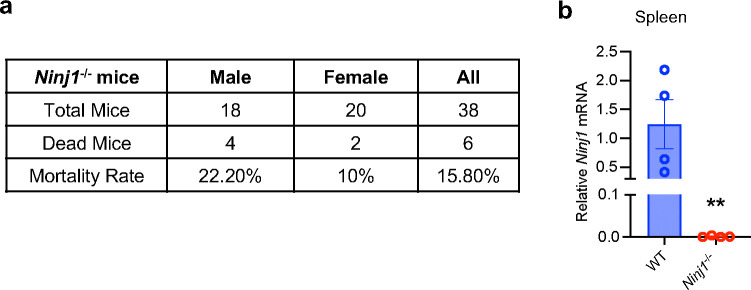
Characterization of Ninj1^−/−^ KO mice. **a**, The mortality rate of the *Ninj1*^−/−^ mice within 2 months from birth. **b**, The relative NINJ1 mRNA levels from the spleen of WT and *Ninj1*^−/−^ mice. n=4 animals per genotype. Data are mean ± s.e.m.. ** p<0.01 vs WT.

To confirm the effect of NINJ1 in HeLa-YFP cells on the stretch-induced quenching response, we transfected the cells with a pool of 4 siRNAs against NINJ1 (SmartPool, to reduce the off-target effects of siRNAs). Consistent with high-throughput screening results, we found that knocking down NINJ1 significantly reduced YFP quenching upon 5-second stretch at 50% strain (Fig. 1j). Trypan Blue staining showed a drastic reduction in blue signals in the cytoplasm in NINJ1 siRNA-treated cells compared to the Scrambled siRNA-treated group (Fig. 1k), indicating fewer PMR events. As expected, LDH release from the NINJ1 knockdown cells after stretch is also significantly lower (Fig. 1l). We further asked if NINJ1 plays a similar role in membrane fragility of cultured primary cells in a more physiologically-relevant setting. We obtained and validated the *Ninj1*^−/−^ mice (Extended Data Fig. 3), then isolated bone marrow-derived monocytes (BMDMs). After maturation, we seeded them onto the stretch membrane and applied 5-second stretch at 45% strain to trigger PMR. Compared to WT BMDMs, which showed ~15% of DRAQ7^+^ cells after stretch, the *Ninj1*^−/−^ BMDMs showed significantly reduced DRAQ7^+^ cells percentage at 5%, indicating a lower PMR rate induced by mechanical strain (Fig. 1m). These results showed that reducing NINJ1 levels renders the plasma membrane more resistant to mechanical stretch.

**Extended Data Fig. 4:**
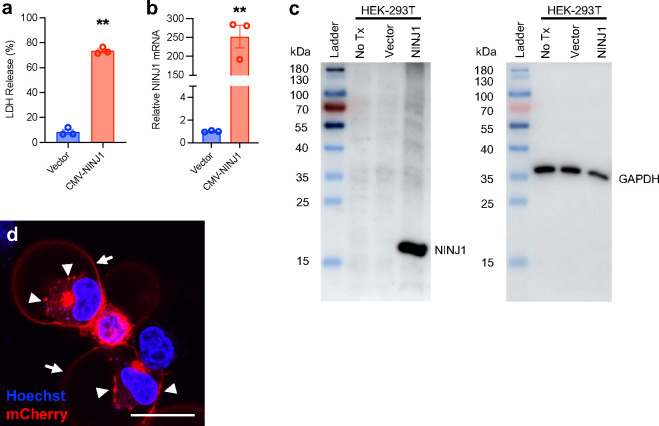
Overexpression of NINJ1 by CMV promoter drastically increases NINJ1 protein level in HEK-293T cells. **a**, LDH release from the vector-transfected HEK-293T cells and the cells overexpressing human NINJ1-IRES-mCherry driven by the CMV promoter. LDH assay was conducted 24 h after transfection. n=3 trials for each group, ** p<0.01 vs Vector. **b**, Relative mRNA level of control HEK-293T cells and CMV-NINJ1 transfected cells at 24 h post-transfection. n=3 for each group. ** p<0.01 vs Vector. **c**, Immunoblot of NINJ1 on an SDS-PAGE gel of HEK-293T cells with no-transfection (no Tx), transfected with vector or CMV-NINJ1-IRES-mCherry. Cell lysate were collected 24 h post-transfection and immunoblotted using a polyclonal human NINJ1 antibody. GAPDH was used as loading control. **d**, Confocal image of HEK cells overexpressing NINJ1-mCherry fusion under the CMV promoter showing high intensity of fluorescence located to the ER and plasma membrane. Fluorescent puncta were visible (arrowheads) and plasma membrane ballooning were evident in some cells (arrows) 24 h post-transfection. NINJ1 was visualized with direct mCherry fluorescence. Nuclei were live stained with Hoechst. Scale bar, 25 μm.

We then asked if increasing NINJ1 expression makes the membrane more fragile and susceptible to rupture by stretch. Consistent with previous reports, overexpression of NINJ1 using a conventional CMV promoter-driven cDNA expression plasmid in HEK-293T cells caused a high level of cytotoxicity^3^. The supernatant LDH level is significantly higher in NINJ1-overexpressing cells compared to vector-transfected controls (Extended Data Fig. 4a). We reasoned that this could be due to the high efficiency of the CMV promoter causing the cells to produce excessive amount of NINJ1 protein, therefore making the plasma membrane extremely vulnerable to even minute mechanical strain caused by normal cell culture handling procedures. Indeed, qPCR showed a 250-fold increase in NINJ1 mRNA level 24h post-transfection (Extended Data Fig. 4b). Immunoblotting on an SDS-PAGE gel showed a dramatic increase of NINJ1 protein level in overexpressing HEK cells (Extended Data Fig. 4c). To visualize overexpressed NINJ1 in the cells, we constructed a CMV-NINJ1-mCherry fusion plasmid and transfected HEK-293T cells. Fluorescence images show that overexpressed NINJ1-mCherry fusion protein mostly located to the plasma membrane and ER, and puncta were clearly visible, with some cells developing ballooned morphology 24h-post transfection (Extended Data Fig. 4d). These data support the notion that excessive NINJ1 expression could disrupt plasma membrane integrity and cause cytotoxicity.

**Fig. 2:**
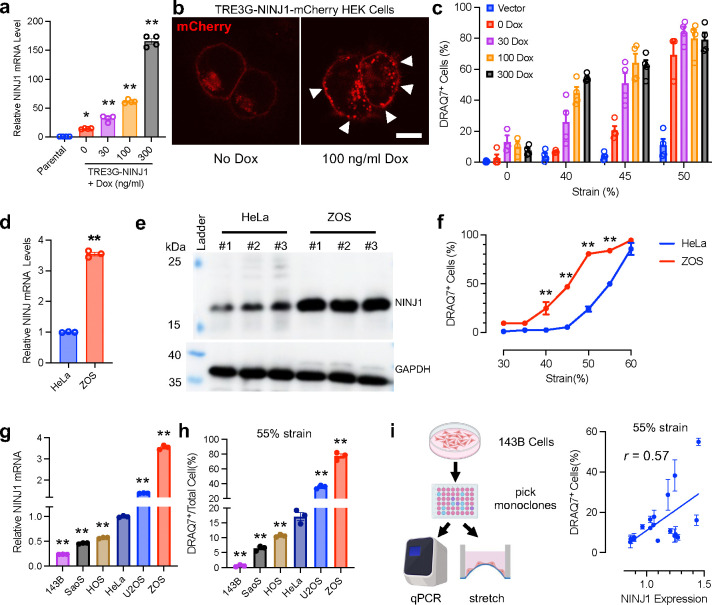
NINJ1 renders the plasma membrane susceptible to rupture under mechanical tension. **a**, Relative NINJ1 mRNA levels of HEK-293T parental cells and the cells stably expressing NINJ1 driven by a Doxycycline-inducible TRE3G promoter. mRNAs were extracted and measured 24 h after induction by Doxycycline of various doses. N=4, ** p< 0.01 vs parental cells **b**, Confocal image of TRE3G-NINJ1-mCherry HEK stable cells with no induction or induction by 100 ng/ml Doxycycline for 24 h. Fluorescent puncta were apparent after induction (arrowheads). Scale bar, 10 μm. **c**, HEK parental cells or TRE3G-NINJ1-mCherry stable cells were treated with Doxycycline at various doses to induce NINJ1 expression, then mechanical strain was applied at 24h post-induction. PMR events were measured by DRAQ7^+^ staining. n=3~4 trials from a total of 300~500 cells per group. **d**, The relative NINJ1 mRNA level of HeLa and ZOS cells were determined by qPCR. 3 trials were conducted for each group. **e**, the immunoblot of NINJ1 from HeLa and ZOS cells on a SDS-PAGE gel. 3 replicates were done for each cell line (The full gel images are in Extended Data Fig. 5). **f**, The percentage of DRAQ7^+^ HeLa and ZOS cells were quantified 30 min after stretches of various strain levels. Stretches were 5s in duration. Data are from 3–6 trials. A total of 300~500 cells were analyzed for each group. ** p<0.01 vs HeLa. **g**, The relative NINJ1 mRNA levels from a collection of the human osteosarcoma cell lines. n=3 trials for each group. ** p<0.01 vs HeLa. **h**, The percentage of DRAQ7^+^ osteosarcoma cell lines were quantified 30 min after stretches at 55% strain. Stretches were 5s in duration. Data are from 3 trials. A total of 300~400 cells per group were analyzed for each group. ** p<0.01 vs HeLa. **i**, Single clones of 143B cells were picked by serial dilution and subjected to mRNA level measurement by qPCR followed by stretch test at 55% strain and DRAQ7 staining. 18 clones were isolated and tested. A total of 300~400 cells were analyzed for each clone. Unless otherwise noted, all data are mean ± s.e.m..

**Fig. 3:**
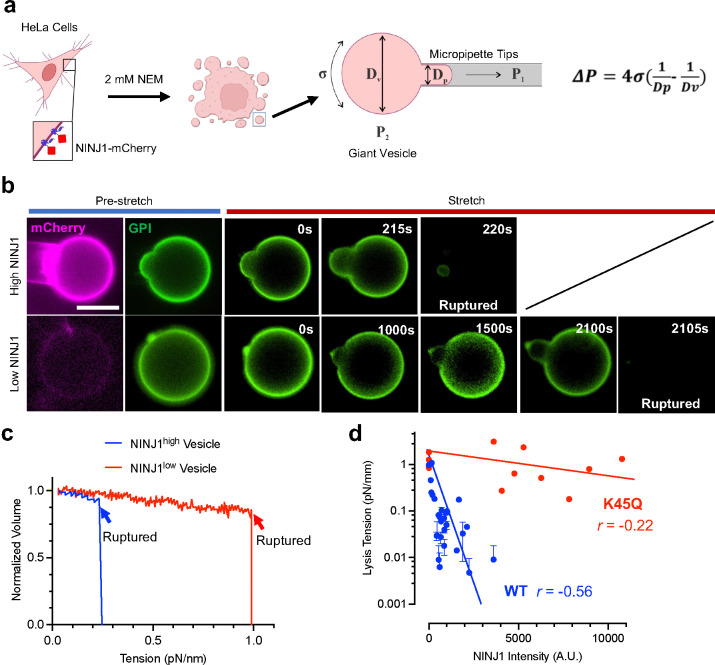
NINJ1 level inversely-correlates with the tension required to rupture the plasma membrane. **a**, HeLa cells expressing NINJ1-mCherry fusion were treated with 2mM NEM to induce giant plasma membrane vesicle (GPMV) formation. A micropipette was used to capture the GPMVs and apply increasing negative pressure until the vesicle ruptures. **b**, GPMVs with high levels of NINJ1 ruptured at lower pressure during aspiration protocol than the ones with low levels of NINJ1. NINJ1 was visualized by fluorescence from mCherry fused at C-terminal of the protein. Fluorescently labeled GPI was used to visualize the membrane. Scale bar, 10 μm. **c**, The trace of a representative NINJ1^high^ vesicle and a NINJ1^low^ one, showing the volume changes during the aspiration protocol. **d**, The correlation of lysis tension of GPMVs and the NINJ1 protein level on the membrane, as indicated by the intensity of mCherry fluorescence. 28 vesicles harboring WT NINJ1, and 13 vesicles with K45Q NINJ1 (a mutated version with reduced activity) were tested.

To investigate the quantitative relationship between NINJ1 expression level and membrane integrity, we engineered a HEK-293T stable cell line harboring a Doxycycline-inducible TRE3G-NINJ1-mCherry construct. This allowed us to modulate NINJ1 expression by controlling the dosage of Doxycycline added to the culture medium, as indicated by mRNA level 24h-post induction (Fig. 2a). With no Doxycycline added, the cells appeared healthy, and low amount of NINJ1-mCherry was observed in ER and plasma membrane due to leaky expression. With 100 ng/ml Doxycycline induction for 24 hours, more mCherry puncta were detected, but the cells remain mostly without herniation (Fig. 2b). We next applied mechanical strain to those cells after the induction by Doxycycline of various doses, and stained DRAQ7 post-stretch to assay PMR events. Cells treated with lower dose of Doxycycline, therefore had lower NINJ1 expression, ruptured at higher strain levels, while the cells with high NINJ1 induced by high Doxycycline dose, ruptured at lower strain levels. In cells treated with 300 ng of Doxycycline, ~60% cells experienced PMR at 40% strain, a level that had no effect on either parental cells, or TRE3G-NINJ1-mCherry stable cells with no Doxycycline treatment (Fig. 3c). This result demonstrates that high NINJ1 levels renders the cells more susceptible to rupture under mechanical tension.

**Extended Data Fig. 5:**
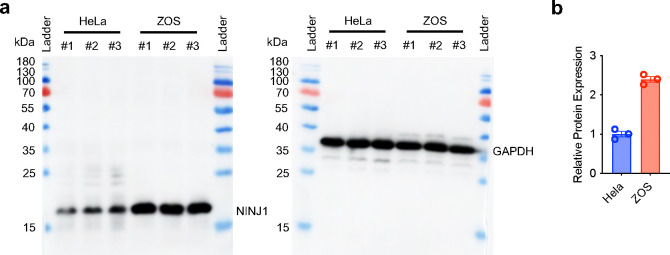
Measurement of relative NINJ1 protein levels in HeLa and ZOS cells. **a**, The full image of the immunoblot of NINJ1 from HeLa and ZOS cells on a SDS-PAGE gel. 3 replicates were done for each cell line. **b**, the quantification of relative protein levels of NINJ1 in HeLa and ZOS cells.

To test if the effect of NINJ1 on plasma membrane fragility under mechanical tension is a general, cell-type autonomous phenomenon, we tested the ZOS cells, an osteosarcoma cell line with higher endogenous NINJ1 mRNA and protein levels compared to HeLa cells (Fig. 2d, e, Extended Data Fig. 5), to see if its plasma membrane is more prone to rupture under tension. Compared to HeLa cells, the PMR rate of ZOS cells was significantly higher compared to HeLa cells after applying 5-second stretch, starting from 40% strain. At 50% strain, the PMR rate was more than 3 times that of HeLa’s, indicating it is indeed more susceptible to PMR under strain (Fig. 2f). We further collected a series of osteosarcoma cell lines with varying mRNA levels of NINJ1, and measured their PMR rate under high strain. We found that the percentage of DRAQ7^+^ cells after the application of 55% strain held the same trend of the respective endogenous NINJ1 mRNA level of the cell lines, confirming that NINJ1 expression level correlates with likelihood of PMR under mechanical strain (Fig. 2g, h).

Different genetic background from various cell lines could result in differences in cytoskeleton organization, extracellular matrix (ECM) production, ECM-binding protein, etc., and this could significantly impact their plasma membrane fragility in a complex way. We reasoned that if we started from one parental cell line and pick single clones derived from it with natural variation of endogenous NINJ1 levels, and measure their PMR rates under tension, it would help further isolate the effect of NINJ1 from other compounding genetic factors. We therefore picked 18 single clones from a pool of 143B cells, measured their mRNA levels and the respective PMR rates under application of 55% strain. Indeed, we found that the susceptibility to rupture of those clones under strain is correlated with their endogenous NINJ1 expression level (Fig. 2i). Taken together, these results show that NINJ1 is a critical, cell type-autonomous regulator of plasma membrane integrity under mechanical strain.

## NINJ1 protein level is inversely-correlated with the tension required to rupture the plasma membrane

On the cellular level, the mechanical integrity of the plasma membrane is influenced by a series of components including the phospholipid bilayer, membrane-integrated proteins and the cytoskeleton, etc. As NINJ1 is a multi-pass membrane protein, we hypothesized that effect of NINJ1 on PMR under mechanical strain could be the result of it modifying the biomechanical properties of the membrane through its interaction with the lipid bilayer. To test this, we employed giant plasma membrane vesicle (GPMV) technique, since in GPMV models, the major components are mostly lipids and transmembrane proteins, and there’s minimal cytoskeleton involved^17^. Briefly, HeLa cells overexpressing NINJ1-mCherry fusion were treated with N-ethylmaleimide (NEM) to induce blebbing, the resulting vesicles were picked up by a micropipette, which applied suction with increasing amplitude until the vesicles ruptured. Lysis tension, the tension within the plasma membrane at the time of rupture, can be derived from recorded parameters (Fig. 3a). We found that with vesicles with low NINJ1 levels, as vacuum suction gradually increases, the membrane inside the pipette deformed slowly, eventually rupturing at high tension (Supplementary Video 3). On the other hand, the vesicles with high NINJ1 levels ruptured at much lower pressure levels (Fig. 3b, c, Supplementary Video 4). The lysis tension of the GPMVs is inversely correlated with the protein level of NINJ1 on the vesicle membrane, suggesting that high NINJ1 renders the plasma membrane more fragile under mechanical tension (Fig. 3d). The vesicles derived from cells expressing an NINJ1-K45Q, a mutant with reduced cytotoxicity^3^, have much higher lysis tension around 0.2 to 2 pN/nm range, and without apparent correlation to the amount of protein expressed (Fig. 3d). These results show that the protein level of NINJ1 is inversely-correlated with lysis tension of the plasma membrane, indicating that NINJ1 regulates membrane fragility under mechanical tension by affect the biomechanical properties of the lipid bilayer directly.

## NINJ1 lowers the mechanical threshold of full plasma membrane rupture in lytic cell deaths

**Extended Data Fig. 6:**
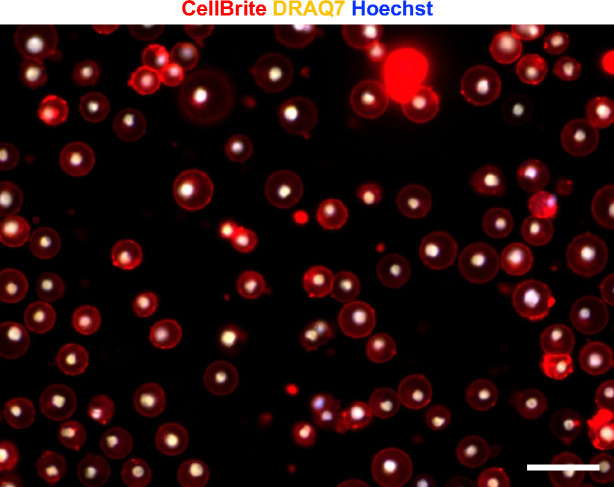
The pyroptotic THP-1 mostly maintain its ballooned morphology after induction of pyroptosis in the absence of mechanical stress stimulation. The THP-1 cells were treated with 5 μg/ml Nigericin and stained for plasma membrane (CellBrite, red), nuclei of cells with compromised membrane (DRAQ7, yellow) and all nuclei (Hoechst, blue) 48 hours later. Cells with permeabilized plasma membrane showed DRAQ7/Hoechst double staining, represented in white. Scale bar, 50 μm.

**Fig. 4:**
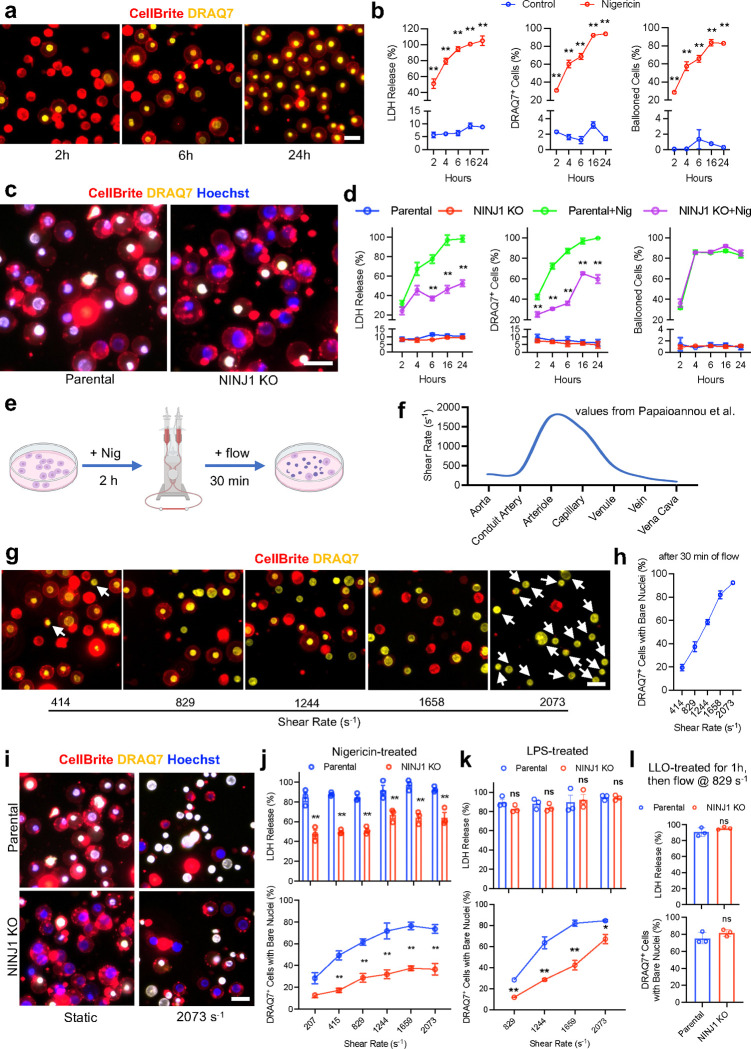
NINJ1 weakens the plasma membrane and facilitate PMR under mechanical stress during lytic cell deaths. **a**, THP-1 cells were treated with 5 μg/ml Nigericin to induce pyroptosis. Plasma membrane was visualized by CellBrite (red). DRAQ7 (yellow) was used to label the nuclei of cells with compromised plasma membrane (permeabilized cells). **b**, LDH release, percentage of DRAQ7^+^ cells, and percentage of the ballooned cells were measured at 2, 4, 6, 16 and 24h after Nigericin treatment. n=3, ** p<0.01 vs control. **c**, Parental and NINJ1 KO THP-1 cell were treated with 5 μg/ml Nigericin. In addition to CellBrite (red) and DRAQ7 (yellow), Hoechst (blue) was used to label all nuclei regardless of the state of the plasma membrane. Permeabilized cells were the ones with DRAQ7^+^/Hoechst^+^ nuclei (represented in white). Non-permeabilized cells displayed Hoechst^+^ nuclei (in blue). **d**, LDH release, percentage of the DRAQ7^+^ and the ballooned cells of parental and NINJ1 KO cells were quantified at 2, 4, 6, 16 and 24h after Nigericin treatment. n=3, ** p<0.01 vs parental. **e**, Work flow of the experiment testing the effect of mechanical stress on PMR in pyroptotic THP-1 cells. **f**, Diagram adapted from Papaioannou et al. showing the estimated shear rate in various vessel beds in humans. **g**, Pyroptotic THP-1 cells were stained with CellBrite (red) and DRAQ7 (yellow), after applying flow stimulation at different shear rates for 30 minutes. Cells were treated with 5 μg/ml Nigericin 2 h before the onset of flow. Arrowheads in the 414 s^−1^ and the 2073 s^−1^ panel denotes the DRAQ7^+^ THP-1 cells with bare nuclei without apparent CellBrite staining surrounding them, indicating the cells with fully ruptured plasma membrane. Arrowheads were not added for other panels to reduce image clutter. **h**, Quantification of full PMR events after applying flow of different shear rates. **i**, Representative images from WT and NINJ1 KO pyroptotic THP-1 cells after applying flow with a shear rate of 2073 s^−1^ for 30 min. Cells with no flow treatment (static) were used as control. Both groups were treated with Nigericin 6h prior to the experiment. Cells with fully ruptured membrane displayed DRAQ7^+^/Hoechst^+^ bare nuclei (in white). **j**, Quantification of the LDH release, percentage of fully ruptured parental and NINJ1 KO cells 30 minutes after the onset of flow with shear rates ranging from 207 s^−1^ to 2073 s^−1^. **k**, Cells were electroporated with 500 ng LPS 1h prior to the stimulation by flow with 829 s^−1^, 1244 s^−1^, 1658 s^−1^ and 2073 s^−1^ in shear rate. LDH release, percentage of fully ruptured parental and NINJ1 KO cells were measured after flow stimulation for 30 min. **l**, Cells were treated with 500 ng/ml LLO for 1h, then subjected to flow at 829 s^−1^ in shear rate. Measurement of the LDH release, percentage of fully ruptured parental and KO cells were done after 30 min of flow stimulation. In all assays, data are from 3 trials. To quantify DRAQ7 staining and ballooned cell percentage, 250~300 cells examined for each trial, ** p<0.01 vs parental. All scale bars are 25 μm.

NINJ1 was first described as an adhesion molecule that is upregulated after nerve injury^18–20^. Recent studies show that NINJ1 is involved in post-death events that allow the full rupture of pyroptotic cells, but the underlying mechanisms remain unclear, possibly through the formation of oligomers of various degrees and altering lipid bilayer structures^3,4,21,22^. Given that we identified NINJ1 through a mechanical force-centric unbiased genetic screen, we tested if physical force is a previously unidentified regulatory factor in lytic cell deaths involving NINJ1. We first treated THP-1 cells, a type of monocyte-like suspension cells, with Nigericin, which induces pyroptosis through activation of canonical inflammasome^23^. As expected, we observed a steady increase in LDH in the supernatant after Nigericin treatment, with the levels reaching the maximum from 6 hours onwards (Fig. 4b). The percentage of DRAQ7^+^ cells followed the same trend, indicating the formations of pores, crevices or tears on the plasma membrane. Remarkably, most of the DRAQ7-stained cells maintained a ballooned morphology without full plasma membrane rupture (Fig. 4a, b, membrane visualized with CellBrite), all the way up to 24~48 hours (Extended Data Fig. 6, Supplementary Video 5). This indicates that in THP-1 cells, after the induction of pyroptosis and the presumptive activation of NINJ1, the gaps, crevices or tears on the plasma membrane are large enough for LDH and DRAQ7 to pass through, however the plasma membrane still have sufficient structural resilience to hold the larger cellular contents, preventing them from leaking out to the extracellular environment.

To further investigate the role of NINJ1 in this pyroptosis setting, we knocked out NINJ1 in THP-1 cells and compared them to the parental cells. We found that NINJ1 KO cells showed about 50% less LDH release at 24 hours post-Nigericin treatment (Fig. 4d). Consistently, the KO cells showed significantly lower percentage of cells with DRAQ7 staining (Fig. 4d). Importantly, both NINJ1 KO and parental cells maintained ballooned shape without significant rate of full rupture events (Fig. 4c, d). These results demonstrate that induction of pyroptosis in THP-1 cells by Nigericin and the presumed NINJ1 activation do not automatically lead to the full rupture of the plasma membrane, suggesting that additional external forces might be needed to exert mechanical strain to achieve full PMR in pyroptotic cells.

To test the role of the external force in this setting, we utilized a commercial laminar flow system to apply mechanical stimulation to pyroptotic THP-1 cells (Fig. 4e). For suspension cells, the shear rate is the relevant measurement of mechanical force being experienced by cells, compared to the wall shear stress which pertains to endothelial cells. *In vivo*, the shear rate generated by flowing blood in various vessel beds range from 10 s^−1^ in Vena Cava to ~2000 s^−1^ in arterioles (Fig. 4f)^24^. We treated THP-1 cells with Nigericin and after 6 hours, applied flow to them at shear rates varying from 415 s^−1^ to 2073 s^−1^, an appropriate range of values to cover most vascular beds under physiologically conditions. After 30 min of flow stimulation at a shear rate of 415 s^−1^ (equivalent to large arteries, large veins and venules), most herniated THP-1 cells maintained the ballooned morphology without full membrane rupture (Fig. 4g). About 20% of the DRAQ7^+^ cells had full rupture and showed bare nuclei, with no apparent ballooned plasma membrane surrounding them (Fig. 4g, arrow). Strikingly, application of flow at a shear rate of 2073 s^−1^ (equivalent to higher end of the values in arterioles) to the same cells resulted in full rupture in 90% of the DRAQ7^+^ cells, with most of the cells show bare nuclei with no surrounding plasma membrane (Fig. 4g). The full rupture rate of pyroptotic THP-1 cells showed a positive association with the shear rate (Fig. 4h). To see how NINJ1 affects full rupture of pyroptotic suspension cells induced by flow, we compared the NINJ1 KO THP-1 cells with the parental cells after treatment with flow ranging from 207 s^−1^ to 2073 s^−1^ in shear rate. After flow stimulation for 30 min, the percentage of parental cells that reached fully ruptured state (represented by Hoechst^+^/DRAQ7^+^ bare nuclei, in white color) increased as the shear rate elevated. The NINJ1 KO cells had significantly lower LDH release, as well as the fewer fully ruptured cells to parental cells. Remarkably, more than 60% of the NINJ1 KO cells maintained ballooned but intact plasma membrane even after flow stimulation as intense as 2073 s^−1^ in shear rate (Fig. 4i, j). These results demonstrated that, during pyroptosis through canonical inflammasome induced by Nigericin, NINJ1 is a critical determining factor of full PMR under mechanical tension.

**Extended Data Fig. 7:**
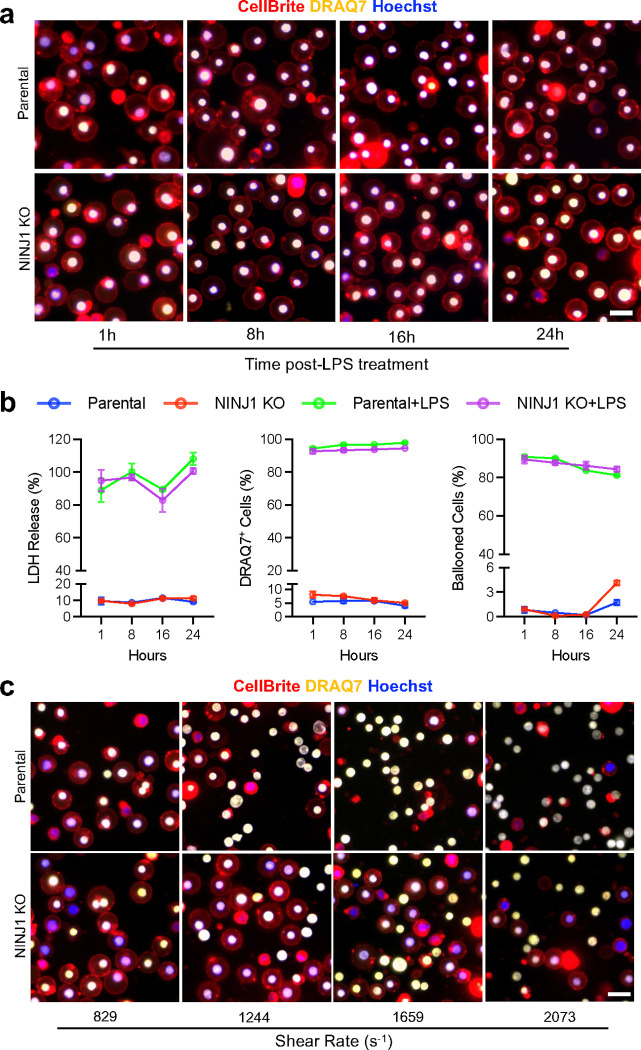
NINJ1 facilitates full PMR under mechanical stress in intracellular LPS induced cell death. **a**, NINJ1 KO and parental THP-1 cells were electroporated with LPS and stained with CellBrite (red), DRAQ7 (yellow) and Hoechst (blue) at 1h, 8h, 16h and 24h after LPS treatment. Almost all cells showed DRAQ7 staining, indicating large gaps or openings on the plasma membrane, yet still displayed intact ballooned shape without full rupture. **b**, Quantification of LDH release, percentage of DRAQ7+ cells and percentage of ballooned cells at various time points after electroporation of LPS. n=3 for each group. No difference was observed between NINJ1 KO and parental cells. **c**, NINJ1 KO and parental THP-1 cells were electroporated with LPS 1 hour before being subjected to flow treatment at various shear rates from 829 s^−1^ to 2073 s^−1^. The cells were stained as previously described. A significantly lower percentage of NINJ1 KO cells show DRAQ7^+^/Hoechst^+^ bare nuclei, indicative of full rupture of the plasma membrane, after being subjected to mechanical stress exerted by fluid flow. Scale bar, 25 μm.

To see if NINJ1 plays a similar role in pyroptotic cell death mediated by non-canonical inflammasome, we electroporated THP-1 cells with lipopolysaccharide (LPS) and measured LDH release and DRAQ7 permeation. We found that after treatment with intracellular LPS, the most of the cells swelled and held the ballooned from 1 hour onwards (Extended Data Fig. 7a). LDH release and the percentage of DRAQ7^+^ cells also reached the maximum level at 1 hour. Interestingly, unlike in Nigericin-induced cell death, there’s no difference between NINJ1 KO and parental cells in terms of LDH release or the percentage of DRAQ7^+^ cells (Extended Data Fig. 7b). When flow was applied after 1 hour of LPS treatment, both the parental and KO cells show full LDH release, as expected. However, NINJ1 KO cells appeared significantly more resistant to mechanical stress exerted by the flow at various shear rate. The percentage of cell reached full PMR state is significantly lower than that of the parental cells across the shear rate range (Fig. 4k). Notably, the biggest difference between the genotypes arises at the shear rate from 1244 s^−1^ to 1659 s^−1^, the equivalent of the mechanical stress that the suspension cells encounter in arterioles and capillaries, suggesting that NINJ1 could be crucial for regulating circulating immune cell rupture in small vessels during inflammation.

**Extended Data Fig. 8:**
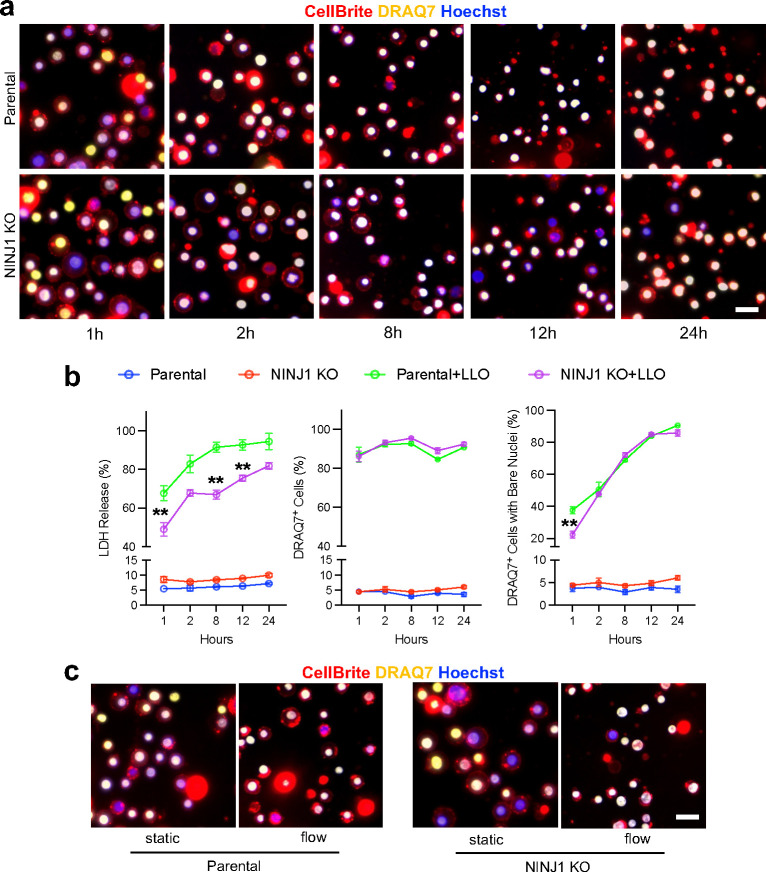
NINJ1 does not significantly affect full PMR under mechanical stress in LLO induced cell death. **a**, NINJ1 KO and parental THP-1 cells were treated with 500 ng/ml LLO and stained with CellBrite (red), DRAQ7 (yellow) and Hoechst (blue) at 1h, 2h, 8h, 12h and 24h later. **b**, Quantification of LDH release, percentage of DRAQ7^+^ cells and DRAQ7^+^ cells with bare nuclei at various time points after LLO treatment. NINJ1 KO cells showed significantly lower LDH release compared to WT in all time points. It also showed significantly fewer cells with bare nuclei (indicative of full ruptured membrane) at 1h post LLO treatment, but was similar to WT from 2h onwards. n=3 for each group. ** p<0.01 vs parental. **c**, NINJ1 KO and parental THP-1 cells were treated with LLO for 1h before being subjected to flow treatment at the shear rate 829 s^−1^. After 30 min of flow stimulation, the cells were stained as previously described. Both KO and parental cells showed high full rupture rate. No significant difference was observed between the genotypes. All Scale bars, 25 μm.

Finally, we investigated the role of NINJ1 in cell deaths induced by direct damage to the plasma membrane, such as those inflicted by pore-forming toxins, e.g., Listeriolysin O (LLO). Treating WT THP-1 cells with LLO led to a significant increase of LDH release from 1 hour onwards. In comparison, NINJ1 KO cells show lower LDH release after the same LLO treatment (Extended Data Fig. 8a, b). This phenotype was similar to that of the mouse primary BMDMs as previously reported^3^. We then treated the cells with LLO and after 1 h, applied flow at 829 s^−1^ for 30 min, and found that both parental and NINJ1 KO cells showed high levels of LDH release and full PMR in a majority of cells (Fig. 4l, Extended Data Fig. 8c). These results show that in cell death caused by direct membrane damage, NINJ1 may not be the major determining factor of full plasma membrane rupture under strain, suggesting that the membrane weakening effect of NINJ1 could be limited to those programmed cell death mediated by intrinsic cellular components.

In summary, our results demonstrate that in programmed cell death, NINJ1 is critical for modifying the biomechanical properties of the phospholipid bilayer and renders the plasma membrane more fragile, but itself is not sufficient for the full rupture of the plasma membrane. Additional mechanical forces are needed to execute full PMR, facilitating the release of DAMPs for downstream signaling processes. Our results suggest that the interplay between NINJ1 activation and mechanical stress provides a mechanism for complex regulation of cell death-associated PMR across tissues with diverse mechanical microenvironments.

## Discussion

In this study, we designed and constructed a custom high-throughput cellular stretch system from the ground up, and through large-scale siRNA screen, we identified NINJ1 as a critical regulator of plasma membrane fragility under mechanical tension. We further demonstrated quantitatively that NINJ1 level is inversely-correlated to the tension required to rupture the membrane. Previous studies showed that NINJ1 could oligomerize into large structures such as filaments, branches or pores, which presumably lead to damages to the plasma membrane^3,21,22^. We suggest that high level of NINJ1 protein, possibly in oligomerized state, could alter the biomechanical properties of the plasma membrane, creating “engineered weak points” within it. However, this may not necessarily lead to membrane rupture on its own, and mechanical forces are needed for the full execution of PMR. Under strain levels that are normally innocuous to a cell with low or no NINJ1, but damaging to a cell littered with NINJ1 oligomers, larger tears may start to appear and propagate in the plasma membrane, eventually reaching a full rupture, akin to a cellular “Tear-Along-Perforation” mechanism. Remarkably, we show that in our stretch experimental settings, NINJ1 regulates PMR without the activation any cell death programs. This suggests that PMR might not a direct consequence of programmed cell death *per se*, but could be an independent post-cell death or cell death-accompanying event, and mechanical force is a crucial player in the PMR process.

Exactly what induces NINJ1 activation or oligomerization is not clear. During program cell death, there are several possibilities might trigger NINJ1 activation. For instance, the changes in phospholipids composition of the lipid bilayer during program cell death could cause the conformational change of the transmembrane segments, particularly in the first two short helix regions, which could drive oligomerization of the NINJ1 monomers. Alternatively, upregulation of NINJ1 expression could be sufficient to induced aggregation by crowding, as we demonstrated in overexpression system driven by the high-efficiency CMV promoter, cells with excessive NINJ1 expression showed more visible puncta on the plasma membrane and ER, possibly leading to weakened membrane, PMR and cytotoxicity. Lastly, the ballooning and stretching of the membrane may bring about curvature and tension changes that could affect interaction between transmembrane segments of the monomers and favor the aggregated form. However, in our GPMV experiments, the mCherry-tagged NINJ1 display a uniform distribution of fluorescence along the membrane, and we did not observe apparent formation of fluorescent puncta while the membrane tension increased, suggesting either tension may not be a causal factor of the puncta formation that we saw in other experiments, or it requires other co-factors that are absent in GPMVs. Due to the optical limitation of the PDMS substrate used in the stretching experiments, super resolution image was not possible, and we were not able to test if increasing membrane strain can induce formation of the puncta in an intact cell. Future studies, possibly utilizing other techniques, such as NMR, are needed to clarify the mechanism of NINJ1 activation.

The biological function of NINJ1 is relatively understudied. It was first identified as an adhesion molecule that is upregulated by sciatic nerve injury and promotes axonal growth^18^. Recently, NINJ1 was shown to be important for inflammatory responses in bacterial infection and several liver injury models in mice, possibly by controlling PMR and the release of DAMPs^3,4,25,26^. Our results show that elevated expression of NINJ1 weakens the plasma membrane and increases PMR probability under mechanical strain. *In vivo*, mechanical strain takes many forms. For instance, blood flow exerts shear stress on monocytes while circulating in the blood vessels. Beating heart and breathing lungs apply tension to various cardiac and pulmonary cell types. Skeletal muscle cells experience tears during local motion^2^. These may have profound effects on PMR of the pyroptotic cells and DAMP release in their respective tissue microenvironment. Whether NINJ1, in conjunction with physical force, plays roles in inflammation modulation in mechanically-active tissue microenvironments warrants further investigation. Interestingly, in the absence of mechanical forces, NINJ1 is required for LDH release and DRAQ7 permeation during Nigericin-induced pyroptosis, whereas its deficiency has no effect on these processes in intracellular LPS-induced pyroptosis (Fig. 4d, Extended Data Fig. 7b). These findings suggest that NINJ1 is not tightly associated with large molecule permeation processes, implying that it may belong to a different class of proteins than established pore-formers like GSDMD. It is plausible that NINJ1 has additional, yet unidentified, biological functions beyond its role in PMR. Notably, NINJ1-deficient animals exhibit developmental hydrocephalus^27^, underscoring the need for careful investigation to fully uncover NINJ1’s functions.

Finally, in this work, we have demonstrated the utility of our novel high-throughput cellular stretch system in identifying novel biology in PMR under mechanical tension. Our system has the potential for broader impact in mechanobiology. With its vastly improved throughput over traditional means, it could be highly useful in identifying the elusive high-threshold mechanosensitive ion channels in DRG neurons that underlie the sensation of mechanical pain in the absence of PIEZO2^28^. It could also help to reveal the molecular identity of channels that mediate the mechanically-activated anion currents. Lastly, it can find success as a drug discovery tool, facilitating the discovery of specific inhibitors of mechanically activated channels and receptors such as PIEZO1/PIEZO2, TMEM63 family ion channels^29^, etc., contributing to the development of novel therapies for disease conditions caused by mechanotransduction defects.

## Methods

### Engineering of the high-throughput cellular stretch system and the consumables

The high-throughput cellular stretch system consists of two major components, the main unit and the executive terminals. The main unit connects to an oil-free vacuum pump (Fujiwara, FUJ-V3) to provide vacuum drive up to −93 kPa. The stepless modulation of vacuum is achieved by using an electronic vacuum regulator (SMC, ITV2090-04N2N5), and the vacuum level is monitored by an in-line high-precision digital pressure switch (SMC, ZSE20A-T-C6H-J). A custom user interface was written using C# and allows the user to set intensity, amplitude and frequency of the stimulation via a touchscreen. The electronic governor performs closed-loop calculations upon receiving commanded vacuum parameters from the user interface, and utilizes the pressure switch’s readings to generate corresponding signals for the vacuum regulator, which functions to achieve stepless control of vacuum in proportion to electrical signals, thereby modulating the levels to match commanded parameters. The real-time vacuum levels are recorded by pressure switch and instantaneously displayed on the interface for the user to monitor running of the system. We designed two types of executive terminals for the main unit, one single-well vacuum head intended to use with microscopes and one whole-plate vacuum manifold intended for plate readers (such as Molecular Devices FLIPR). To construct the body of single-well head, a 50 mm diameter brass cylinder stock was cut to 15 mm in heigh using an electric bandsaw, and was manually machined on a lathe to level out the top and bottom surfaces. A 4.0 mm through whole was drilled in the center and a quick-connect push fitting (SMC, KQ2R04-06) was glued in by epoxy. The brass cylinder then was plasma treated and two silicone rubber rings (25 mm O.D.−15 mm I.D. and 12 mm O.D.−3.5 mm I.D.) were affixed to the bottom surface (stem-side of the fitting). The head connects to the main unit using a 4.0 O.D. polyurethane tubing (SMC, TU-0425). The weight of the head assembly (~280 g) helps seal the wells when resting on top of the assay plate.

The whole plate manifold was manufactured on a CNC bed mill (Kent Industrial USA, KVR-4020A) using aluminum alloy. A 1/8” threaded hole was added to the center of the manifold and an 8 mm O.D. quick-connect push fitting (SMC, KQ2H08-U01A) fitted. A baffle insert was added to the interior of the manifold to smooth out the air for better aerodynamics. Six locator tabs were incorporated to the frame to facilitate the alignment of the manifold to the assay plate. All aluminum parts were anodized to a matte black finish, and fasteners painted black to reduce light scattering in the imaging chamber of the plate reader. A silicone rubber seal was frictionally fitted to the grove machined in the frame to allow easy disassembly for cleaning and maintenance. Stretchable PDMS-bottom 384-well assay plates were developed and manufactured in house from bottomless 384-well plate blanks (Greiner Bio-One, 781000-06). The plate blanks were plasma treated for 5 min, 50% power at a vacuum of 0.2 mBar using a plasma surface treater (Plasma Technology GmBH, SmartPlasma 10). Following this, a 200 μm-thick optically-clear PDMS membrane (Guangzhou Ehang Electronics) was affixed to the bottomless 384-well plate using pressure-sensitive adhesive. The plates were stored in dark at room temperature. Before each use, the plates were exposed to ultraviolet irradiation in the biosafety cabinet (NuAire, NU-543) for 30 minutes.

### Estimation of the strain by finite element analysis

To gain a better understanding of the membrane deformation in 3D, we used FEA (Finite Element Analysis) to model the assay plate with stretchable membrane under vacuum using SimScale CAE software (SimScale GmbH). Only one well was modeled as a representation of the entire plate to assist in computational speed. A second order finite mesh with 15657 tetrahedral elements and 28268 nodes was generated with an automatic mesh generator. The PDMS membrane material was considered incompressible, having a Young’s modulus of E = 1.40 MPa, Poisson’s ratio ν = 0.49, and density ρ = 1120 kg/m^3^. The bottomless 384-well polystyrene assay plate blank adhered to the PDMS membrane was modeled with a Young’s modulus of E = 1.8 GPa, Poisson’s ratio ν = 0.35, and density ρ = 1200 kg/m^3^. Fixed support boundary conditions were applied to the exterior walls of the single well assay plate to represent the connecting, adjacent well walls in a full 384-well assay plate. To simulate the various vacuum levels, negative pressure conditions ranging from −10 to −40 kPa were assigned to the top surface of the PDMS membrane inside the well and the simulation was executed and results saved.

### Estimation of the strain by particle imagery analysis

The particle imagery method was used for estimating the strain of PDMS membrane on the bottom of the assay well. Briefly, we dispensed a suspension (0.01% w/v) of green fluorescent polystyrene beads of 5 μm diameter (Tianjin BaseLine, 7-3-0500, Ex 488nm, Em 518nm) into the wells of the stretch plates and let them settle and adhere to the bottom. On an inverted fluorescence microscope (Olympus, BX63F), we first located the center of the well and captured still images of beads at resting, non-stretched state, and recorded the Z-axis position. We then applied vacuum and held at designated intensities, captured the bead positions and recorded their Z-axis positions at stretched state. Strain levels of the PDMS membrane were estimated using the following formula:

$$Strain(\%)\approx \left(\frac{\sqrt{d^{\prime 2}\;+\;h^{2}}}{d}-1\right)\times 100$$

Where d is the distance of a given bead from the center of the well at the resting state, $d^{’}$ is the distance at the stretched state. d and $d^{’}$ were measured from images take before and during the stretch respectively. h is the height difference of between the bead and the top center of the stretched PDMS dome formed under vacuum, and was derived from Z-axis position readings supplied by the microscope stage controller.

### Animals

All animals used in this study were maintained under specific pathogen-free conditions and cared for in accordance with National Institutes of Health guidelines. All experiments involving animals were carried out with experimental protocols and procedures reviewed and approved by the Institutional Animal Care and Use Committee of Sun Yat-sen University (SYSU-IACUC-2023-000575).

*Ninj1*^−/−^ mice were purchased from GemPharmatech (Nanjing, China, Catalog number: T034357). We obtained homozygous knockout mice (*Ninj1*^−/−^) by intercrossing heterozygous mice and compared them with wild-type littermates in all experiments. About 15.8% of the *Ninj1*^−/−^ mice develop hydrocephalus and die after 4 weeks of age (Extended Data Fig. 3a). *Ninj1*^−/−^ mice with no hydrocephalus syndrome (large head and low body weight) at 12–16 weeks old were used in the experiments. Knockout of *Ninj1* has been confirmed in spleen by qRT-PCR (Extended Data Fig. 3b).

### Plasmids, chemicals and reagents

Complementary DNA (cDNA) for human NINJ1 and NINJ1 K45Q mutant^3^ were purchased from BGI Genomics. The NINJ1 and NINJ1 K45Q mutant cDNAs were inserted into a CMV promoter vector with PVAT-mCherry for transient expression in HEK-293T cells and HeLa cells and the pLVX TRE3G vector with PVAT-mCherry for stable expression in HEK-293T cells, respectively.

The plasmid pCDNA3.1-EYFP-H148Q-I152L was obtained from pCDNA3.1-Ano1-m-T2A-EYFP-H148Q-I152L (P1668) through homologous recombination to construct HeLa-YFP cells^15^. Cell culture products were all purchased from GIBCO. Poly-D-Lysine was used (Thermo Fisher Scientific, A3890401) for 384-well plate coating. High iodine solution for YFP quenching assay contains 100 mM NaI (Macklin, S817516–100g), 40 mM NaCl (Aladdin, C111535–500g), 5 mM KCl (Aladdin, R100832–250mg), 1 mM CaCl_2_ (Sigma-Aldrich, C5670–100g) and 20 mM HEPES (Solarbio, H1095–100) with pH adjusted to 7.4 using NaOH (LEAD-BIO, LD-8013–100ml). Chloride channel inhibitor DCPIB was purchased from Cayman Chemical (34064–5mg). SiRNAs were purchased from Genepharma (A10001), Lipofectamine^™^ RNAiMAX (Invitrogen, 13778–150), FuGENE^®^ HD Transfection Reagent (Promega, E2311) and Opti-MEM (Thermo Fisher Scientific, 31985070) were used for transfection of cultured cells. 5 μm-diameter green Lumisphere fluorescent beads (BaseLin) were used for imagery analysis to derive the strain of PDMS membrane under vacuum. Nigericin (Selleck, S6653) was used to induce cell pyroptosis. Doxycycline (Selleck, S5159) was used for the induction of TRE3G-regulated plasmid expression. CytoTox 96 Non-Radioactive Cytotoxicity Assay (Promega, G1780) for LDH assay. Hoechst (Thermo Fisher Scientific, 62249) for cell nucleus staining. Trypan Blue (GIBCO, 15250061) and DRAQ7^™^ Dye (Invitrogen, D15106) to indicate loss of plasma membrane integrity and cell death. CellBrite^®^ Fix 555 (Biotium, 30088) were used for labeling cell plasma membrane. Stocks of chemicals were reconstituted in DMSO (AAT Bioquest, ST038) and stored at −20 °C unless stated otherwise. Nigericin were dissolved in 100% ethanol and stored at −80 °C. LLO were stored at −80 °C.

### DNA and siRNA Transfection

Cells were plated into a 24-well plate, reaching a cell confluence of approximately 70–80% before transfection. For transient plasmid transfection, FuGENE^®^ HD Transfection Reagent was used following the manufacturer’s instructions. Plasmids was introduced into the culture system at a concentration of 1 μg/ml. For stable expression, lentiviral plasmids harboring the desired gene were first transfected into HEK-293T cells together with the packing plasmids pSPAX2 and pMD2G with a ratio of 4:3:1. The supernatants were collected 48 h after transfection and used to infect HEK-293T cells for another 48 h. mCherry-positive infected cells were sorted by flow cytometry (BD Biosciences FACSAria II). For siRNA transfection, Lipofectamine^™^ RNAiMAX was used at the recommended concentrations according to the manufacturer’s instructions. siRNA was added to the culture system at a concentration of 20 pM.

### Cell Culture

HeLa YFP cells were isolated as single-cell-derived clones through flow cytometry sorting following transfection with the halide-sensitive YFP (with H148Q and I152L mutations) plasmid. 293T, HeLa were purchased from ATCC (CCL-2). Osteosarcoma cell lines, including ZOS, ZOSM, U2OS, 143B, HOS, SJSA, and SaoS, were kindly provided by the Dr. Changye Zou and Dr. Caixia Xu. Wildtype THP-1 cells were purchased from Cell Bank of Chinese Academy of Sciences. All the cell lines are frequently checked by morphological features and functionalities, but have not been subjected to authentication by short tandem repeat (STR) profiling. All the cell lines have been regularly tested for the presence of mycoplasma by a detection kit (Vazyme, D101–02). To obtain single-cell-derived clones of the 143B cell line, parental cells were dissociated from culture vessels and made into single cell suspension, then sorted into individual wells of a 96-well plate using a cell sorter (BD FACSAria^™^ Fusion) and subsequently cultured for two weeks prior to further study.

HeLa, HEK-293T and the aforementioned osteosarcoma cell lines were grown cultured in Dulbecco’s Modified Eagle’s Medium (DMEM) (GIBCO, C11330500BT) containing 4.5 mg/ml glucose, supplemented with 10% fetal bovine serum (FBS) (ExCell Bio, FND500) and 1% penicillin-streptomycin (PS) (GIBCO, 15140122). THP-1 cells were maintained in RPMI-1640 medium (GIBCO, C22400500BT) supplemented with 10% FBS, 1% penicillin-streptomycin, 0.05 mM 2-Mercaptoethanol (Sigma-Aldrich, M3148–25). For experiment, THP-1 cells were cultured in RPMI 1640 supplemented with 1% fetal bovine serum, 1% penicillin-streptomycin and then plated at approximately 2 × 10^5^ cells ml^−1^ in 150 μl in 96-well plates. For pyroptosis induction, THP-1 cells were treated with Nigericin (5 μg/ml for WT THP-1 cells and 20 μg/ml for engineered NINJ1 KO and the corresponding parental cell, Selleck, S6653) and analyzed at various time points. Supernatant were collected for LDH assay. All media were supplemented with 10% (vol/vol) fetal bovine serum (FBS) and 2 mM L-glutamine. All cells were kept in a humidified incubator set at 37 °C with 5% CO_2_, with medium refreshment carried out every other day.

### YFP Quenching Assay

HeLa-YFP cells in 384-well plate were washed and maintained in 30 μl/well wash solution (in mM): 140 NaCl, 5 KCl, 1 CaCl_2_ and 20 HEPES, with pH adjusted to 7.4 using NaOH. 2 min before the start of the stretch experiment, a 30 μl/well high iodide solution (in mM) (100 NaI, 40 NaCl, 5 KCl, 1 CaCl_2_ and 20 HEPES, with pH adjusted to 7.4 using NaOH) was added to the wells. A 20~30 second baseline fluorescence was recorded before applying stretch stimulation, with recording continuing for 2 min. The YFP fluorescence 2 s before the stretch stimulation ($F_{0}$) and at the end of recording (F) were used to quantify the response to the stretch stimulation, termed Quenching Score, as follows^15^:

$$Quenching\;Score=\frac{\left|F\;-\;F_{0}\right|}{F_{0}}$$

### Assay of stretch-induced cell death and PMR

Cells were seeded in a Poly-D-Lysine-coated PDMS-bottom 384-well plate, achieving approximately 70–80% confluence before experimentation. For cells harboring genes driven by TRE3G inducible promoter, doxycycline (Selleck, S5159) at specified concentration was added to the culture medium to induce gene expression. The cells were then incubated with 1.6 μM Hoechst 33342 (Invitrogen) and 1.5 μM DRAQ7 Dye (Invitrogen, D15106) in 1x Hanks Balanced Salt Solution (HBSS) for 15 minutes at room temperature. Following the incubation, stretch stimulation was applied, and cells were imaged using an inverted fluorescence microscope (Olympus) 2 hours later. Imaging was performed in the DAPI, Cy5, and bright field channels. Image analysis was carried out with OlyVia software and ImageJ.

### High throughput siRNA screening

The 384-well PDMS stretch plates were labeled with a microplate labeler (Agilent, G5581AA) to facilitate data processing and management. 24 h prior to siRNA transfection, plates were coated with poly-D-lysine (Advanced BioMatrix, 5049). Coating was done by dispensing 10 μL 0.1 mg/ml poly-D-lysine solution into each well by a high-throughput robotic liquid handler, and incubate at room temperature for 2 h. The plates were then washed with ddH_2_O and air-dried in a biosafety cabinet (NuAire, NU-543) for 6 h then kept in a 4 °C cold room overnight. One the day of transfection, siRNA library was arrayed from master plates to stretch plates using an automated liquid handler (Bravo, Agilent) by transferring 1 μl of 20 μM siRNA stock solution for each well. A master mix of Lipofectamine RNAiMAX/Opti-MEM (ratio: 8 μL/1 mL) was made and dispensed to the wells at 25 μl/well and incubated at room temperature for 20 min. Afterwards, 25 μl of the HeLa-YFP cell suspension (450k cells/ml in 20% FBS) were dispensed into each well. The plates were shaken at 6,000 rpm for 10 s on microplate shaker and incubated at 37 °C with 5% CO_2_. The next day, a medium change was carried out by taking out 35 μL old medium and replenish with 60 μL fresh medium. 72 h post-transfection, the cells were washed three times with 1×HBSS on a microplate washer (BioTek, ELx450CW), and moved to assay stretch-induced YFP quenching on a plate reader (Molecular Devices, FLIPR Tetra). A 30 s baseline fluorescence was recorded at 1 frame every second, and a single stretch of 5 s in duration at 50% strain was applied to the cells, while the FLIPR was continuously recording. 60 s later, the recording frequence was reduced to 1 frame every 5 s until the recording ended at 150 s (or manually stopped as required). Raw data was acquired and data sequence files were exported using ScreenWorks 4.2.1.2 (Molecular Devices) for further analysis. The YFP intensity at end of the recording (150 s) was first normalized to the mean intensity of the whole plate, under the assumption that most siRNAs will not have effects on the quenching response, yielding a value $F_{\text{normalized}}$ for each well. Well values from all plate were then spliced together to form an overall dataset, then a mean of all data points ($F_{\text{overall\_mean}}$) and standard deviation ($SD_{\text{overall}}$) were derived. A Z-Score for each well, representing how far away this well is from the bulk of the data, was calculated as follows:

$$Z_{-}Score=\frac{F_{\mathrm{normalized}}\;-\;F_{\mathrm{overall\_mean}}}{SD_{\mathrm{overall}}}$$

We used the Z-Score >1.5 as the cut-off line for the primary screen data, yielding a list of 755 primary hit siRNAs. We then collected siRNAs against these genes for a reconfirmation screen, further reducing the size of the hit list by applying more stringent cut-off line. From the second round of screen onwards, genes were required have multi-hits, i.e., two or more siRNAs working, to be selected to go forward. In the final round, experiments were carried out using siRNAs against 20 genes purchased from a separate source (from a retail catalog instead of from a library catalog of Dharmacon) to increase the confidence of the screen.

### Western blots

Samples were lysed in RIPA buffer (Beyotime, P0013E) with Protease inhibitor PMSF (Beyotime, ST506) at 4 °C for 30 min. Supernatants were collected after centrifuge at 13,800 g at 4 °C for 30 minutes and the protein concentration was measured (Beyotime, P0010). 10 μg protein was mixed with SDS-PAGE sample buffer (Beyotime, P0286) and run in 12.5% SDS–PAGE gel (EpiZyme, PG113). Human NINJ1 antibody (1:200, R&D Systems, AF5105), anti-human GAPDH antibody (1:10000, Proteintech, 60004–1-LG), HRP-conjugated Goat Anti-Mouse IgG (1:5000, Proteintech, SA00001–1), HRP-conjugated Rabbit anti-Sheep IgG (1:5000, Abclonal, AS023) was used for blotting. The gel was visualization by the FDbio-Dura ECL chemiluminescence kit (Fdbio science, FD8020). The gel images were acquired by a CCD imager (Cytiva, Amersham ImageQuanta 800). Images were quantified using ImageJ v1.52p grayscale analysis.

### Quantitative reverse transcriptase PCR

For qRT-PCR analysis, total RNA was extracted using the EZ-press RNA Purification Kit (EZBioscience, B0004D). Subsequently, cDNAs were generated with the PrimeScript RT Reagent Kit (Takara, RRO47A). RT-qPCR was performed using TB Green Premix Ex Taq II (Takara, RB820B) and on a real-time PCR system (ThermoFisher Scientific, QuantStudio). The PCR conditions included an initial denaturation step at 95 °C for 30 seconds, followed by 40 cycles of amplification at 95 °C for 5 s and 55~60 °C for 30 s. The mRNA expression of target genes was assessed, with 18S serving as the internal reference gene. Relative expression levels were calculated using the 2^−ΔΔCt^ method^30^.

### Bone marrow-derived macrophage (BMDM) isolation and culture

Primary BMDM cells were prepared from 8~12-week-old mice (C57BL/6 background, both gender) by following a standard procedure as previously described^31,32^. For each experiment, four mice were pooled to prepare the BMDM cells for assaying the cell death responses. Bone marrow cells from wild-type or *Ninj1*^−/−^ mice were differentiated into macrophages in DMEM supplemented with 10% (v/v) fetal bovine serum (FBS, Capricorn Scientific) and macrophage colony stimulating factor (M-CSF, day0: 10 ng/ml; day3: 20 ng/ml, PeproTech, 315–02) at 37 °C, 5% CO_2_ for 7 days.

### GPMV preparation and Micropipette Aspiration on GPMVs

HeLa cells (ATCC, CCL-2) were grown in DMEM High Glucose medium (Gibco, 11965118) supplemented with 10% FBS and 1% penicillin-streptomycin to approximate 50–70% and transfected with plasmids coding hNINJ1-mCherry fusion protein using TransIT-X2 (Mirus Bio, MIR 6004). 24 h after the transfection, cells were washed with PBS twice and GPMV buffer (10 mM HEPES, 150 mM NaCl, 2 mM CaCl_2_, pH7.4) twice^17^. Then the transfected cells were incubated with 2 mM N-ethyl maleimide (NEM, Pierce, 23030) in GPMV buffer for 2 hours at 37 °C. After 2 hours of incubation, the supernatant was transferred to a glass bottom dish (Cellvis, D35–14-1.5-N) which was coated with 1 mg/ml bovine serum albumin (Gibco, 16170086) for 15 minutes at room temperature.

Micropipettes were prepared by pulling borosilicate glass capillaries (WPI, TW100–4) using Micropipette Puller (WPI, PUL-1000). Pulled micropipettes were then cut and bent by Microforge Controller (WPI, DMF1000). PEG-Silane (Gelest, 65994–07-2) was used to modify the micropipettes’ surface to remove possible attachments of the membrane. Pressure in micropipettes was controlled by microfluidic system (FLUIGENT, ESORT-PCK01), which was manipulated remotely by OxyGEN software. GPMVs were aspirated and lifted from the bottom of the dish by the micropipettes before starting the experiments. During the experiments, pressure was increased stepwise at a rate of 10 Pa per 30 seconds until the GPMVs broke. The lysis tensions of GPMVs were calculated accordingly.

### Generation of NINJ1 KO THP-1 cells

Generation of knockout cell lines by the CRISPR/Cas9 technology was described previously^32^. Briefly, two guide RNAs, 5’-GCTGGGGCCCTGTTCCACGA-3’ & 5’-ATGGAGATGAGGACCACCAG-3’ for *NINJ1*, were cloned into the guide RNA-expression plasmid lentiGuide-Puro (Addgene, 52963). Transient transfection was performed using the JetPRIME kit (Polyplus) following the manufacturer’s instructions. For stable expression, lentiGuide-Puro or lentiCas9-Blast (Addgene, 52962) were plasmids firstly transfected into 293T cells together with the packing plasmids psPAX2 (Addgene, 12260) and pMD2.G (Addgene, 12259) at a ratio of 5:3:2. The supernatants were collected 48 h after transfection and used to infect THP-1 through centrifugation at 800g for 90 min in the presence of polybrene. After approximately 18 h of incubation, all infected cells were centrifuged, and medium was changed to the fresh complete medium. The cells were selected by 2 μg/ml puromycin or 30 μg/ml blasticidin prior to flow sorting. 5 d post-infection, puromycin or blasticidin-resistant live cells were sorted into single clones using a cell sorter (BD FACSAria II). The single clones were cultured in 96-well plates for another 10~14 d (or a longer time depending on the cell growth rate). Genotype of the knockout cells was determined by Sanger sequencing.

### LDH release assay

LDH in cell culture supernatant were analyzed using the CytoTox 96 Non-Radioactive Cytotoxicity Assay (Promega, G1780). The samples’ absorbance at 490nm was measured by a microplate reader (ThermoFisher Scientific Varioskan LUX). LDH release was normalized to untreated and 100% lysis control. LDH release was calculated as follows:

$$LDH\;Release\;(\%)=\frac{LDH_{\mathrm{sample}}\;-\;LDH_{\mathrm{negative\_control}}}{LDH_{\mathrm{full\_lysis}}\;-\;LDH_{\mathrm{negative\_control}}}\times 100$$

### THP-1 staining and imaging

THP-1 cells were pre-stained with CellBrite Fix 555 (1:1000, Biotium, 30088) to visualize cell plasma membrane. Following the pyroptosis induction, the THP-1 cells were stained with DRAQ7 Dye (1:200) and Hoechst 33342 (1:1000). Cells were imaged with an inverted fluorescence microscope (Olympus, BX63F). Images were taken in the preset TRITC, DAPI, Cy5 and bright field channels. Image analysis was performed in OlyVia and ImageJ software.

### Microscopy imaging of cell death

To examine membrane and nucleus morphology, cells were treated as indicated for static image capture or time-lapse imaging with an inverted fluorescence microscope (Olympus, BX63F). All image data shown were representative of at least three randomly selected fields. Time-lapse imaging was captured with the same microscope with the optional environmental controller and gas mixer to maintain cells at 37 °C and 5% CO_2_. Video recording started upon addition of Nigericin and was recorded with 1 min intervals overnight. Manual focal adjustments were carried out as needed throughout the imaging process.

### Estimation of the shear rate

The estimation of the shear rate in the flow experiment using Ibidi system was based on following assumptions: 1, the culture medium is an ideal Newtonian fluid with constant viscosity; 2, the flow is steady and laminar; 3, the elasticity of the plastic syringe and silicone tube is negligible. Under these conditions, the velocity of the fluid in the center of the tube is highest, where its velocity is zero at the surface of the tube wall (no-slip condition). Therefore, the velocity gradient, or shear rate $\dot{\gamma }$ is:

$$\dot{\gamma }=\frac{du}{dr}$$

where u is the fluid velocity and r the radius of the tube^24^. Poiseuille’s law is applied to determine the shear rate as follows:

$$\dot{\gamma }=32\frac{Q}{\pi \;\cdot \;d^{3}}$$

where Q is the volumetric flow rate and d the tube diameter. The instrument-indicated flow rate, the converted volumetric flow rate and the shear rate calculation used in the current study is listed in Extended Data Table 1.

### Fluid flow stimulation of THP-1 cells

Flow-induced shear stress stimulation of THP-cells was carried out using the pneumatic-driven flow system (Ibidi, 10902) with the paired RED tubing set (Ibidi, 10962). THP-1 cells were transferred into the flow chamber using a large-orifice transfer pipette and suspended in culture medium to a density of approximately 3×10^5^ cells/ml. For experiments with WT THP-1 cells, 5 μg/ml Nigericin was used to treat the cells for 2 h prior to the onset of the flow. For NINJ1 KO and the corresponding parental cells, 20 μg/ml Nigericin was used to treat the cells for 6 h before flow stimulation. All flow stimuli were 30 min in duration. Cells were imaged for morphology analysis using various membrane and nuclei stains, and supernatant were collected for further experiments.

### LPS Electroporation

To stimulate noncanonical inflammasome activation, LPS was electroporated into THP-1 cells using Neon^™^ Transfection System (Invitrogen) and Neon^™^ Transfection System 100 μL Kit (Invitrogen, MPK10096) following the manufacturer’s instructions. Briefly, 2 × 10^6^ cells were transfected with 500 ng LPS (O111:B4, Sigma, 2630) by double pulse (1300 V, 10 ms). After the delivery of the electric pulse, cells were immediately transferred into prepared culture plate or flow chamber containing prewarmed RPMI 1640 with 1% FBS without PS. Cells were imaged and supernatant were collected for LDH assay at different time points. For mechanical stimulation assay, 1 h after the LPS electroporation, flow was applied to THP-1 at different shear rates in the Ibidi flow chamber for 30 minutes.

### LLO treatment

THP-1 cells were treated with 500 ng/ml LLO (Abcam, ab83345) and analyzed at different time points. Cell supernatants were collected for LDH assay. For mechanical stimulation assay, 1 hour after the LLO treatment, flow was applied to THP-1 at different shear rates for 30 minutes.

### Statistics and Data Analysis

Unless otherwise stated, data were expressed as mean ± s.e.m.. All data sets were analyzed using GraphPad Prism (Version 8.0). Unpaired Student’s *t*-test was used for 2 group comparisons. For data with multiple groups, one-way ANOVA or 2-way ANOVA was performed followed by Bonferroni corrections. Estimation of association was determined by Pearson Correlation Coefficient *r* value. A 2-tailed p-value <0.05 was considered to be statistically significant.

## Extended Data

**Extended Data Table 1:** Calculation of shear rate generated by the Ibidi flow system.

Indicated Flow Rate (ml/min)	Volumetric Flow Rate (m^3^/s)	Tubing Diameter (m)	Shear Rate (s^−1^)
10	1.66667×10^−7^	1.6×10^−3^	414.676221
20	3.33333×10^−7^	1.6×10^−3^	829.352442
30	5×10^−7^	1.6×10^−3^	1244.02866
40	6.66667×10^−7^	1.6×10^−3^	1658.70488
50	8.33333×10^−7^	1.6×10^−3^	2073.3811

The red tubing set was used with the Ibidi flow controller. The inlet and outlet of the tubing set were directly connected with a coupler, without a chambered slide installed.

## Data Availability

Datasets generated during and/or analyzed during the current study are available from the lead contact on reasonable request. Source data are provided with this paper.
